# The mechanisms of class 1A PI3K and Wnt/β-catenin coupled signaling in breast cancer

**DOI:** 10.1042/BST20220866

**Published:** 2023-07-20

**Authors:** Samuel J. Rodgers, Christina A. Mitchell, Lisa M. Ooms

**Affiliations:** Department of Biochemistry and Molecular Biology, Biomedicine Discovery Institute, Monash University, Clayton, VIC, Australia

**Keywords:** breast cancer, INPP4B, phosphoinositide, phosphoinositide 3-kinase, Wnt/β-catenin

## Abstract

The class IA PI3K signaling pathway is activated by growth factor stimulation and regulates a signaling cascade that promotes diverse events including cell growth, proliferation, migration and metabolism. PI3K signaling is one of the most commonly hyperactivated pathways in breast cancer, leading to increased tumor growth and progression. PI3K hyperactivation occurs via a number of genetic and epigenetic mechanisms including mutation or amplification of *PIK3CA*, the gene encoding the p110α subunit of PI3Kα, as well as via dysregulation of the upstream growth factor receptors or downstream signaling effectors. Over the past decade, extensive efforts to develop therapeutics that suppress oncogenic PI3K signaling have been undertaken. Although FDA-approved PI3K inhibitors are now emerging, their clinical success remains limited due to adverse effects and negative feedback mechanisms which contribute to their reduced efficacy. There is an emerging body of evidence demonstrating crosstalk between the PI3K and Wnt/β-catenin pathways in breast cancer. However, PI3K exhibits opposing effects on Wnt/β-catenin signaling in distinct tumor subsets, whereby PI3K promotes Wnt/β-catenin activation in ER^+^ cancers, but paradoxically suppresses this pathway in ER^−^ breast cancers. This review discusses the molecular mechanisms for PI3K–Wnt crosstalk in breast cancer, and how Wnt-targeted therapies have the potential to contribute to treatment regimens for breast cancers with PI3K dysregulation.

## Introduction

The phosphoinositide 3-kinases (PI3Ks) are a family of lipid kinases that phosphorylate the 3′-hydroxyl position of the phosphoinositide inositol ring to generate phosphatidylinositol 3-phosphate (PI(3)P), phosphatidylinositol 3,4-bisphosphate (PI(3,4)P_2_) or phosphatidylinositol 3,4,5-trisphosphate (PI(3,4,5)P_3_). The class IA PI3Ks are heterodimeric complexes, consisting of a catalytic subunit (p110α, p110β, p110δ) and a regulatory subunit (p85α, p85β, p55α, p50α, p55γ), which function downstream of receptor tyrosine kinase (RTK) activation. In the absence of growth factors, the inter-Src homology 2 (iSH2) and N-terminal SH2 (nSH2) domains of the regulatory subunit prevent activation of the catalytic subunit [1]. This is mediated by interactions between the regulatory subunit iSH2 domain and catalytic subunit C2 domain [2], and binding of the regulatory subunit nSH2 domain with the C2, helical, and kinase domains of the catalytic subunit [3–5]. Activation of RTKs such as epidermal growth factor receptor (EGFR), insulin receptor or Met recruits the regulatory subunit to the plasma membrane by binding of its SH2 domains to RTK consensus auto-phosphorylation sites [6]. This promotes allosteric activation of the catalytic subunit which phosphorylates the 3′-hydroxyl position of phosphatidylinositol 4,5-bisphosphate (PI(4,5)P_2_), a phosphoinositide located on the inner leaflet of the plasma membrane, thereby generating the lipid signaling molecule, PI(3,4,5)P_3_.

**Figure 1.**
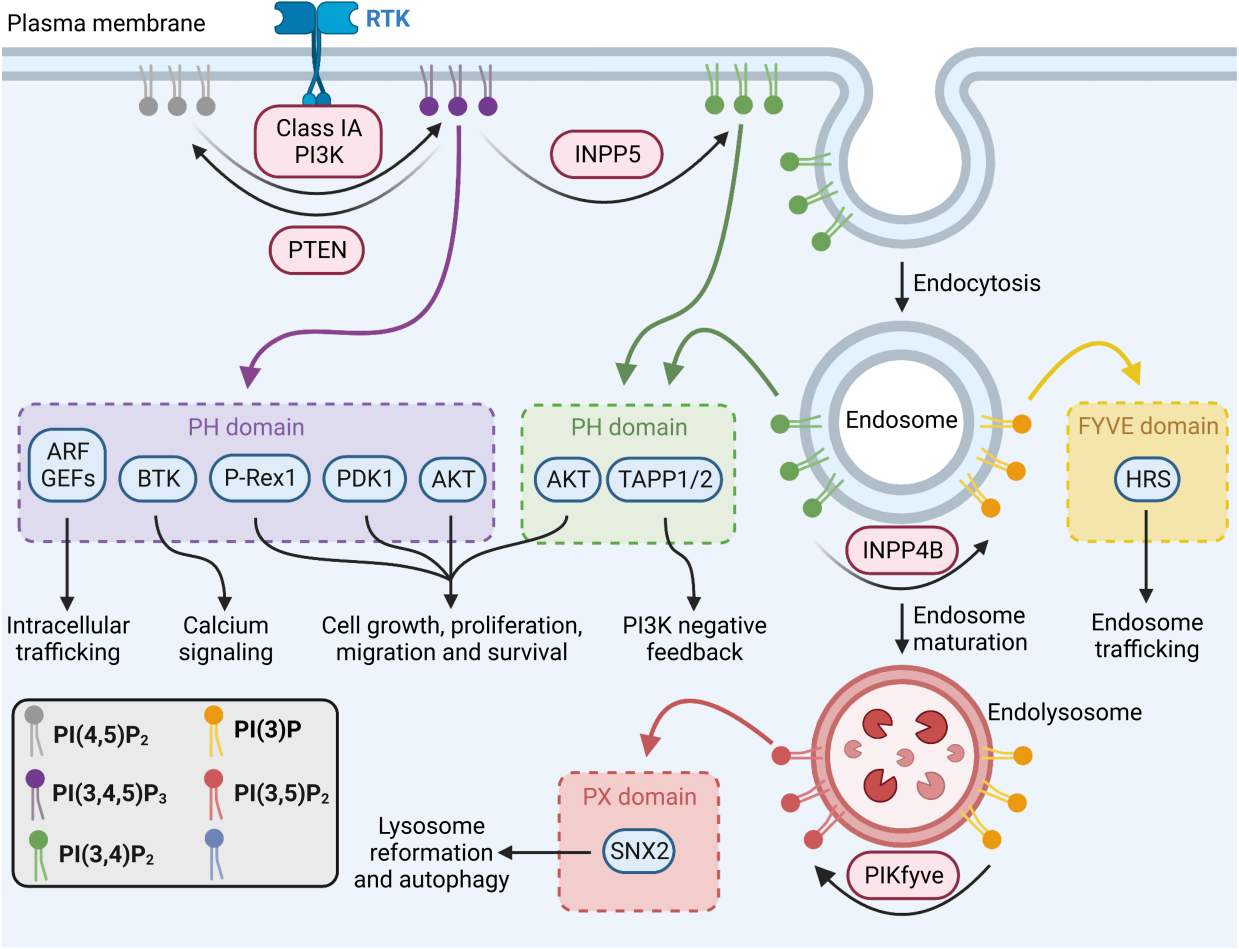
Phosphoinositide-binding effectors of class IA PI3K signaling. Following activation of RTKs, class IA PI3Ks are recruited to the inner leaflet of the plasma membrane where they phosphorylate PI(4,5)P_2_ to generate PI(3,4,5)P_3_. PTEN dephosphorylates PI(3,4,5)P_3_ to PI(4,5)P_2_ and opposes PI3K signaling. PI(3,4,5)P_3_ is also dephosphorylated by inositol polyphosphate 5-phosphatases (INPP5) to generate PI(3,4)P_2_, which is either dephosphorylated by PTEN to PI(4)P, or internalized by endocytosis and further dephosphorylated to PI(3)P by INPP4B. PI(3)P is retained as endosomes mature into endolysosomes and is then subsequently phosphorylated to PI(3,5)P_2_ by PIKfyve. PI(3,4,5)P_3_ and PI(3,4)P_2_ bind and activate numerous PH domain-containing proteins to regulate cell growth, proliferation, migration, survival, intracellular trafficking, calcium signaling or PI3K negative feedback. PI(3)P recruits the FYVE domain-containing protein, HRS, to regulate endosomal trafficking. PI(3,5)P_2_ recruits the PX domain-containing protein, SNX2, to promote lysosome reformation and autophagy. Created with BioRender.com.

PI(3,4,5)P_3_ regulates a diverse network of signaling cascades (recently reviewed [7,8]), by recruiting a number of pleckstrin homology (PH) domain-containing proteins to the plasma membrane (Figure 1). This includes the ADP ribosylation factor guanine nucleotide exchange factors (ARF GEFs) ARF nucleotide-binding site opener (ARNO), cytohesin-1 and general receptor for phosphoinositides 1 (GRP-1) that regulate intracellular membrane trafficking, Bruton's tyrosine kinase (BTK) that promotes calcium signaling, as well as phosphoinositide-dependent kinase 1 (PDK1), mammalian target of rapamycin complex 2 (mTORC2) and phosphatidylinositol-3,4,5-trisphosphate dependent Rac exchange factor 1 (P-Rex1) that promote cell growth and proliferation [9–14]. PI(3,4,5)P_3_ is also the precursor for additional signaling lipids via sequential dephosphorylation and phosphorylation events (Figure 1). PI(3,4,5)P_3_ is dephosphorylated to PI(4,5)P_2_ by phosphatase and tensin homolog (PTEN), which terminates PI3K signaling. PI(3,4,5)P_3_ is also dephosphorylated by inositol polyphosphate 5-phosphatases to generate PI(3,4)P_2_ [15]. Both PI(3,4,5)P_3_ and PI(3,4)P_2_ are required for the plasma membrane recruitment and maximal activation of the serine/threonine kinase AKT, which promotes cell growth, proliferation, migration and survival [16,17]. PI(3,4)P_2_ also recruits tandem PH domain-containing protein 1/2 (TAPP1/2) to suppress PI3K signaling via a negative feedback loop [18]. PI(3,4)P_2_ can be dephosphorylated to PI(4)P by PTEN on the plasma membrane, which suppresses AKT activation [19,20]. PI(3,4)P_2_ is also internalized from the plasma membrane by endocytosis where it facilitates AKT2 activation on endosomes and promotes the endocytic trafficking of RTKs [21–23]. PI3K-dependent endosomal PI(3,4)P_2_ is converted to PI(3)P by inositol polyphosphate 4-phosphatase type II (INPP4B), which recruits the PI(3)P-binding endosomal sorting complex required for transport (ESCRT) protein hepatocyte growth factor-regulated tyrosine kinase substrate (HRS) to promote endosome maturation and protein trafficking [21,24,25]. INPP4B also activates the PX domain-containing protein serum and glucocorticoid-regulated kinase 3 (SGK3) that promotes cell proliferation and invasion [26], though whether this occurs via generation of plasma membrane or endosomal PI(3)P remains unclear. INPP4B-generated PI(3)P is retained as endosomes mature into endolysosomes where it is subsequently phosphorylated to phosphatidylinositol 3,5-bisphosphate (PI(3,5)P_2_) by PIKfyve, which recruits the PI(3,5)P_2_-binding effector sorting nexin 2 (SNX2) to promote lysosome reformation and autophagy [27]. Thus, class 1A PI3K signaling controls diverse cellular processes by recruiting phosphoinositide-binding proteins which in turn activate distinct effectors on subcellular membranes.

## PI3K hyperactivation drives breast cancer initiation and progression

Hyperactivation of class IA PI3K signaling is one of the most common molecular events in human cancers and occurs in more than 70% of human breast cancers [28]. *PIK3CA*, the gene encoding the p110α catalytic subunit of PI3Kα, is mutated in 20–40% of breast cancers, and most frequently in estrogen receptor-positive (ER^+^) breast cancers [29,30]. *PIK3CA* gene amplification also occurs less commonly in ∼9% of breast cancers [31]. The most common *PIK3CA* mutations are gain-of-function missense mutations that occur at two hotspot regions; H1047R in the kinase and E542K/E545K in the helical domain. *PIK3CA^H1047R^* promotes constitutive p110α plasma membrane association [32] whereas *PIK3CA^E542K^* or *PIK3CA^E545K^* mutations preclude p110α binding to the inhibitory p85 subunit [3]. *Pik3ca^H1047R^* conditional mammary knock-in promotes *de novo* tumor formation with long latency and incomplete penetrance [33,34], suggesting *PIK3CA* mutations are weakly oncogenic in isolation. A subset of *PIK3CA*-mutant cancers harbor multiple copies of mutant *PIK3CA* in cis (same allele) or trans (separate alleles) that additively affect downstream signaling and cellular phenotypes in a dose-dependent manner [35–37] or elicit biphasic effects [35–38]. In particular, double *PIK3CA* mutations in cis occur in 8–13% of breast cancers, which enhance cell proliferation and tumor growth compared with single hotspot mutations [35]. Single or multiple *PIK3CA* mutations do not independently correlate with human breast cancer prognosis when corrected for ER-positivity or other favorable-risk variables [35,39].

Class IA PI3K signaling hyperactivation in breast cancer also occurs via other mechanisms including the dysregulation of upstream RTKs that activate PI3Ks, or the lipid phosphatases that regulate downstream phosphoinositide signaling. EGFR is rarely amplified or mutated in breast cancer, but increased gene copy number due to polysomy can occur in triple negative breast cancers, leading to activation of multiple signaling pathways including PI3K [40]. The RTK Met is frequently up-regulated in triple negative breast cancers, and mice with mammary-specific transgenic expression of mutationally activated *Met* develop tumors with moderate penetrance and long latency [41]. Germline *PTEN* mutations cause Cowden disease, which predisposes individuals to tumor development in multiple tissues including breast, thyroid, renal, endometrial and brain [42,43]. *PTEN* is also sporadically deleted or mutated or exhibits promoter hypermethylation in all breast cancer subtypes [44,45]. *Pten^+/−^* mice develop a range of tumors in multiple organs including mammary, prostate, intestine, endometrial and lymphatic tissues [46–49]. Interestingly, PTEN-deficient prostate, colorectal and triple negative breast cancers exhibit dependence on PI3Kβ rather than PI3Kα signaling [50–53]. *Pten^G129E^* (lipid phosphatase-dead) or *Pten^C124S^* (lipid and protein phosphatase-dead) mutant knock-in mice are also prone to mammary, adrenal and thyroid tumors [54]. Proline-rich inositol polyphosphate 5-phosphatase (PIPP), which hydrolyzes PI(3,4,5)P_3_ to PI(3,4)P_2_, exhibits decreased expression in triple negative breast cancers, associated with reduced relapse-free and overall survival [55]. *Pipp* ablation accelerates mammary tumor initiation and growth but reduces metastasis in *PyMT* breast cancer model mice [55]. Loss of heterozygosity (LOH) of the chromosomal region encoding the *INPP4B* gene (4q31.21) occurs in ∼55% of triple negative breast cancers [56,57]. Murine *Inpp4b* knockout enhances mammary tumor incidence in mammary-specific *Tp53^−/−^;Brca1^−/−^* mice and enhances thyroid tumor formation and metastasis in *Pten^+/−^* mice [21,23,58]. However, INPP4B expression is increased in a subset of ER^+^ breast cancers where it paradoxically promotes cell proliferation and tumor growth [25]. Interestingly, *PIK3CA* mutations frequently co-occur with *PTEN* mutations or INPP4B overexpression, which accelerate tumor development [25,59].

There has been significant interest in developing PI3K-targeted therapies for breast cancer due to the high prevalence of PI3K pathway hyperactivation. However, the clinical efficacy of PI3K inhibitors for breast cancer remains limited, as these therapies can elicit significant adverse effects. In addition, feedback signaling mechanisms can reduce drug efficacy over a sustained treatment period. Early inhibitors targeted multiple PI3K isoforms, but few of these successfully progressed through clinical trials. Further clinical progress has been made via specifically targeting the PI3Kα isoform. The PI3Kα inhibitor, alpelisib, was initially found to up-regulate ER-dependent transcriptional activity in *PIK3CA*-mutant ER^+^ breast cancer xenografts, which was circumvented by co-treatment with the selective estrogen receptor degrader (SERD), fulvestrant [60]. Alpelisib was recently approved for advanced *PIK3CA*-mutant ER^+^ breast cancer in combination with fulvestrant, which prolongs relapse-free survival but does not prevent disease recurrence [61]. Studies have identified a number of feedback mechanisms that limit alpelisib efficacy, including PI3K reactivation by p110β in HER2-amplified and *PIK3CA*-mutant breast cancers [62]. In addition, increased pancreatic insulin secretion drives alpelisib resistance in murine mammary tumor models which is overcome by dietary and pharmacological approaches that reduce insulin signaling and restore alpelisib sensitivity [63]. Acquired alterations in other PI3K signaling components can circumvent alpelisib efficacy, such as allelic *PTEN* loss [64]. A current focus is to identify compounds that show higher selectivity for mutant PI3Kα *versus* the wild-type protein, such as inavolisib which promotes HER2-dependent degradation of mutant PI3Kα [65].

## PI3K crosstalk with the Wnt/β-catenin pathway

**Figure 2.**
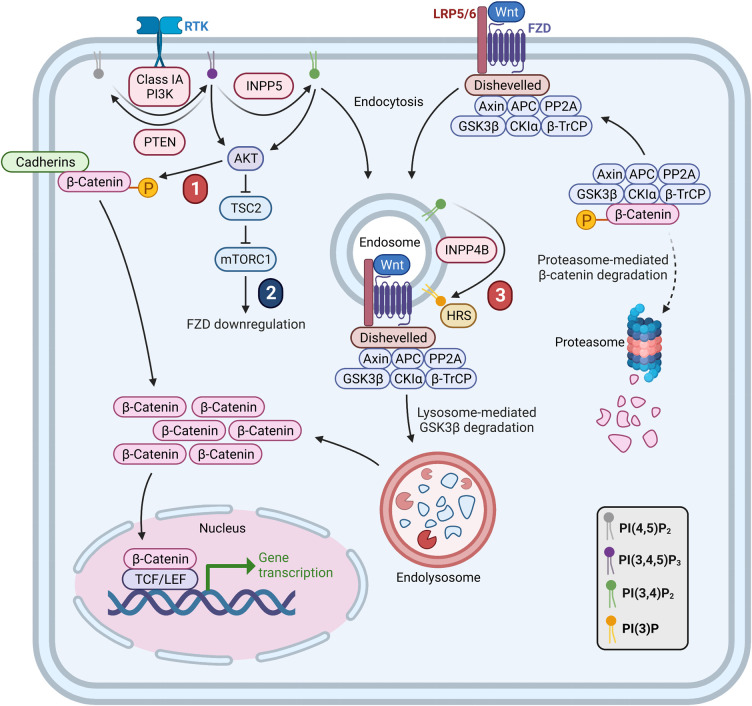
Proposed mechanisms of PI3K and Wnt/β-catenin crosstalk. In the absence of Wnt stimulation, β-catenin is phosphorylated at its N-terminus by the β-catenin destruction complex (AXIN, APC, PP2A, GSK3β, CK1α, β-TrCP) leading to its proteasome-mediated degradation. Wnt ligands bind to FZD/LRP receptor complexes on the plasma membrane, which recruits Dishevelled that in turn recruits the β-catenin destruction complex. Activated FZD/LRP receptors are internalized via endocytosis, which leads to the sequestration of the β-catenin destruction complex within endosomes and lysosome-mediated degradation of GSK3β, allowing activated-β-catenin to accumulate. β-catenin translocates into the nucleus, where it binds to TCF/LEF transcription factors to promote Wnt target gene expression. PI3K is proposed to undergo crosstalk with Wnt/β-catenin signaling via at least three mechanisms: (1) AKT phosphorylates the serine 552 residue of β-catenin at cellular junctions, leading to its accumulation in the cytosol and translocation to the nucleus. (2) AKT phosphorylates and inactivates TSC2, which alleviates the inhibitory effect of TSC2 on mTORC1 leading to mTORC1 activation and down-regulation of FZD levels. (3) INPP4B-generated PI(3)P downstream of PI3Kα facilitates HRS-dependent endosome maturation, which promotes GSK3β endosomal sequestration and lysosomal degradation leading to β-catenin activation. Created with BioRender.com.

The canonical Wnt/β-catenin pathway regulates cell proliferation, migration and differentiation and is hyperactivated in many human malignancies, particularly colon cancer (reviewed in [66]). The central regulatory component of canonical Wnt/β-catenin signaling is the β-catenin destruction complex, which consists of protein phosphatase 2A (PP2A), the protein kinases glycogen synthase kinase 3 beta (GSK3β) and casein kinase 1 alpha (CK1α), the scaffolding proteins AXIN and adenomatous polyposis coli (APC), and the E3 ubiquitin ligase beta-transducin repeat-containing protein (β-TrCP). When Wnt/β-catenin signaling is inactive, CK1α and GSK3β phosphorylate the N-terminal region of β-catenin, leading to its subsequent ubiquitination by β-TrCP and its proteasomal degradation [67] (Figure 2). Wnt/β-catenin signaling is activated via the binding of Wnt ligands to a plasma membrane heterodimeric receptor complex consisting of the seven transmembrane receptor Frizzled (FZD) and the co-receptor low density lipoprotein receptor-related protein 5/6 (LRP5/6) [68,69]. Upon ligand binding, Dishevelled is recruited to the FZD/LRP receptor complex, which promotes FZD/LRP clustering and AXIN binding to facilitate the recruitment of the destruction complex [70,71]. Activated FZD/LRP receptor complexes are internalized by endocytosis leading to the sequestration of the destruction complex within endosomes, which prevents the N-terminal phosphorylation, ubiquitination and degradation of β-catenin [72–74]. As a result, activated-β-catenin translocates into the nucleus where it binds T-cell factor/lymphoid enhancer-binding factor (TCF/LEF) transcription factors to promote Wnt target gene transcription [75].

The PI3K and Wnt/β-catenin pathways share several core signaling components and it has long been predicted that crosstalk exists between these pathways (reviewed in [76]). However, as both PI3K and Wnt/β-catenin networks exhibit a high degree of complexity with numerous feedback loops and context-dependent signaling dynamics [77–79], dissecting the mechanisms of PI3K–Wnt crosstalk has been challenging. Multiple independent studies show that activation of PI3K signaling by insulin stimulation alone is insufficient to concomitantly activate Wnt/β-catenin signaling [80,81]. This is further complicated by the multifaceted role of GSK3β, a core component of the β-catenin destruction complex, which is also independently inactivated via phosphorylation of its Ser9 residue by AKT [82,83]. This has led to speculation that AKT-mediated inactivation of GSK3β promotes Wnt/β-catenin signaling. However, the GSK3β pool bound to the destruction complex is protected from AKT phosphorylation, as its molecular arrangement within the complex shields its Ser9 residue from phosphorylation by AKT [80]. *Gsk3b^S9A^* knock-in mice, which harbor a mutation in the AKT-dependent Ser9 phosphorylation site of GSK3β, show no difference in Wnt3a-dependent activation of β-catenin or Wnt gene transcription [84], revealing that AKT phosphorylation of GSK3β does not significantly contribute to Wnt/β-catenin signaling.

There is growing evidence that PI3K/AKT signaling regulates Wnt/β-catenin signaling independently of GSK3β (Figure 2). A pool of β-catenin is present at cell–cell contacts, where it binds to cadherins and α-catenin to stabilize cellular junctions [85]. AKT directly phosphorylates Ser552 of β-catenin, which enhances β-catenin binding to 14-3-3ζ resulting in β-catenin removal from cellular junctions [86,87]. Phosphorylated β-catenin^S552^ accumulates in the cytosol and nucleus where it promotes Wnt gene transcription and cell invasion [87]. Conditional murine *Pten* inactivation increased AKT activation and nuclear phosphorylated β-catenin^S552^ in intestinal stem cells, leading to enhanced cell proliferation and intestinal polyposis [88]. High levels of PI(4,5)P_2_, the precursor for PI3K generation of PI(3,4,5)P_3_, promotes β-catenin release from VE-cadherin junctions and its nuclear translocation in endothelial cells, whereas PI3K/PI(3,4,5)P_3_ signaling also promotes AKT-dependent β-catenin transcriptional activation of Notch effectors [89]. Endothelial cell deletion of *Inpp5k*, an inositol polyphosphate 5-phosphatase that hydrolyzes both PI(4,5)P_2_ and PI(3,4,5)P_3_, increased β-catenin-dependent *Dll4*/*Notch* transcription leading to defective endothelial tip cell specification and impaired embryonic angiogenesis [89]. Thus, PI3K signaling activates β-catenin transcriptional activity via its removal from cellular junctions, independent of the FZD/LRP receptor and β-catenin destruction complex.

Interestingly, PI3K/AKT signaling also suppresses Wnt/β-catenin signaling via the activation of mTORC1 (Figure 2). AKT phosphorylates and inactivates TSC2, which alleviates the inhibitory effect of TSC2 on mTORC1 leading to its activation [90]. Murine *Tsc2* deletion, which enhances mTORC1 activity, decreased Wnt/β-catenin signaling by reducing cell surface FZD levels and LRP6 phosphorylation in intestinal stem cells, leading to reduced stemness [91]. *FZD* mRNA levels were unchanged following treatment with the mTORC1 inhibitor, RAD001, suggesting that mTORC1 signaling regulates FZD protein degradation, rather than its gene expression [91]. Thus, PI3K has the potential to activate and suppress Wnt/β-catenin via its regulation of distinct downstream effectors. This dichotomy is also observed in different breast cancer subsets, where PI3K promotes Wnt/β-catenin activation in ER^+^ breast cancers, but suppresses this pathway in ER^−^ breast cancers, as discussed in more detail below.

## PI3K signaling enhances Wnt/β-catenin activation in ER^+^ breast cancer

Wnt/β-catenin signaling is hyperactivated in greater than 50% of human breast cancers [92]. Mutations in Wnt pathway components (eg APC, AXIN1/2, β-catenin) occur frequently in other cancers, but are rarely detected in breast cancer suggesting other mechanisms likely contribute to Wnt activation in this context (reviewed in [66,93]). Increased nuclear β-catenin accumulation is observed more frequently in primary human triple negative breast cancers [94,95], whereas ER^+^ breast cancers exhibit an RNA profile consistent with enhanced Wnt/β-catenin signaling [96].

AKT is a central effector of class I PI3K signaling, however, *PIK3CA*-mutant ER^+^ breast cancers show little AKT activation compared with alterations in other PI3K pathway components, and frequently exhibit AKT-independent tumor growth [97,98]. Several independent groups have undertaken RNA profile analysis of primary human breast cancers and mouse models of *PIK3CA* mutant ER^+^ breast cancer and uncovered AKT-independent downstream signaling events that contribute to tumor progression [25,96,99]. *PIK3CA* mutant ER^+^ breast cancers exhibit an RNA expression profile of enhanced Wnt/β-catenin signaling, including up-regulation of Wnt target genes (*AXIN2*, *LEF1*, *MYCN*) and other components such as transcriptional regulators (*TCF7L1*, *TCF7L2*, *CTNNB1*), receptors (*FZD4, FZD7*) and ligands (*WNT5A*). Mammary glands of mice with transgenic expression of human *PIK3CA^H1047R^* or *PIK3CA^E545K^* exhibit increased activated-β-catenin levels compared with wild-type mice [99]. Activated-β-catenin and *AXIN2* mRNA levels are also increased in ER^+^ mouse mammary tumor cells expressing human *PIK3CA^H1047R^* compared with transgenic MMTV-*Her2*/*neu* mouse mammary tumor cells [99]. However, it is unknown whether multiple *PIK3CA* mutations affect Wnt/β-catenin signaling in a dose-dependent or biphasic manner. Treatment with buparlisib (PI3Kα/β/δ/γ inhibitor) reduced the expression of Wnt target genes *AXIN2*, *LEF1*, and *MYCN* in human *PIK3CA*-mutant ER^+^ breast cancer cells [25]. Thus, PI3K signaling promotes activation of Wnt/β-catenin signaling in ER^+^ breast cancer (summarized in Table 1), although whether other class IA PI3K isoforms contribute to Wnt activation in this context remains unclear. The Tankyrase1/2 inhibitor XAV939, which stabilizes the β-catenin destruction complex and suppresses Wnt/β-catenin signaling, sensitized *PIK3CA^H1047R^* expressing mammary tumor cells derived from a doxycycline inducible-*PIK3CA^H1047R^* mouse to LY294002, a non-selective PI3Kα/δ/β inhibitor that also inhibits other PI3K-related kinases as well as unrelated proteins, although little effect was observed in mammary tumor cells derived from *PIK3CA^H1047R^* transgenic mice or *PIK3CA^H1047R^* ER^−^ breast cancer cells for unknown reasons [99].

**Table 1:** Summary of PI3K and Wnt/β-catenin crosstalk in breast cancer

PI3K signaling aberration	Effect on Wnt signaling	Crosstalk mechanism	Effect of Wnt inhibition
**ER^+^ breast cancer**			
Mutant *PIK3CA*	*PIK3CA*-mutant ER^+^ primary human breast cancers exhibited increased Wnt target gene, receptor and ligand mRNA expression [25,96]. *PIK3CA^H1047R^* expression increased activated-β-catenin and Wnt target gene expression in ER^+^ mouse mammary tumor cells [99]. *PIK3CA^H1047R^* or *PIK3CA^E545K^* expression increased activated-β-catenin in mouse mammary glands [99].	Not reported.	XAV939 (TNK1/2 inhibitor) enhanced cytotoxic effect of LY294002 (inhibitor of PI3Kα/δ/β and other proteins) in *PIK3CA^H1047R^*-expressing mouse mammary tumor cells [99].
INPP4B overexpression	INPP4B overexpression increased activated-β-catenin and Wnt target gene expression in *PIK3CA*-mutant ER^+^ human breast cancer cells [25,102].	INPP4B generates PI(3)P on endosomes, which promotes Hrs-dependent endosomal sequestration and lysosomal degradation of GSK3β, leading to Wnt/β-catenin activation [25].	LGK-974 (PORCN inhibitor) rescued enhanced proliferation of INPP4B-overexpressing *PIK3CA*-mutant ER^+^ human breast cancer cells [25]. Pyrvinium (CK1α inhibitor) reduced viability and 3D spheroid growth of INPP4B-overexpressing *PIK3CA*-mutant ER^+^ human breast cancer cells [102].
**ER^−^ breast cancer**			
Buparlisib (PI3Kα/β/δ/γ inhibitor)	Buparlisib treatment increased PORCN, Wnt ligand and receptor mRNA expression in ER^−^ human breast cancer cells [106].	Not reported.	LGK-974 (PORCN inhibitor) and buparlisib treatment synergistically reduced the viability and xenograft tumor growth of ER^−^ human breast cancer cells [106].
Pictilisib (strong PI3Kα/δ, modest PI3Kβ/γ inhibitor)	Pictilisb treatment increased LRP6 phosphorylation, β-catenin nuclear translocation and Wnt target gene expression in ER^−^ human breast cancer cells [107].	Not reported.	LGK-974 (PORCN inhibitor) and pictilisib treatment synergistically reduced ER^−^ human breast cancer cell proliferation [107].
Gedatolisib (dual PI3Kα/γ and mTOR inhibitor)	Not reported.	Not reported.	Cofetuzumab pelidotin (PTK7 inhibitor) enhanced anti-tumor effects of gedatolisib in phase I trials of individuals with ER^−^ breast cancer [113].

INPP4B, which converts PI(3,4)P_2_ to PI(3)P downstream of PI3Kα, contributes to PI3K-dependent activation of Wnt/β-catenin signaling in ER^+^ breast cancer. Although INPP4B functions as a tumor suppressor in triple negative breast cancer by suppressing PI(3,4)P_2_-mediated AKT activation [21,56,57], INPP4B mRNA and protein expression are up-regulated in primary human *PIK3CA*-mutant ER^+^ breast cancers, enhancing tumor cell proliferation and growth via activation of Wnt/β-catenin signaling or SGK3 activation [25,26]. Wnt/β-catenin activation facilitates the endocytosis of the FZD/LRP receptor complexes and the sequestration of the β-catenin destruction complex within endosomes [72,100,101]. INPP4B localizes to early endosomes in a variety of cell types including mouse fibroblasts and thyroid cancer cells [22,23]. In *PIK3CA*-mutant ER^+^ breast cancer cells, INPP4B localizes prominently to late endosomes via its interaction with the small GTPase Rab7 [25]. This interaction enhances PI(3,4)P_2_ conversion to PI(3)P and thereby promotes HRS-dependent endosome maturation and cargo trafficking [25]. As a consequence, INPP4B overexpression increases the endosomal sequestration and lysosomal degradation of GSK3β, leading to increased activated-β-catenin and Wnt target gene transcription [25] (Figure 2). Intriguingly, INPP4B increased SGK3 and Wnt/β-catenin signaling despite suppressing AKT activation. However, whether INPP4B expression affects AKT-dependent β-catenin^S552^ phosphorylation has not been reported. The porcupine O-acyltransferase (PORCN) inhibitors LGK-974 or IWP-2, which prevent Wnt ligand secretion, rescued the increased proliferation of INPP4B-overexpressing ER^+^ breast cancer cells [25]. INPP4B-overexpressing *PIK3CA*-mutant ER^+^ breast cancer cells were selectively sensitive to nanomolar concentrations of pyrvinium [102], an FDA-approved anthelmintic drug that binds and activates CK1α to suppress Wnt/β-catenin signaling [103], and were significantly more sensitive to pyrvinium in combination with the standard-of-care treatment 4-hydroxytamoxifen (4-OHT) [102]. Further investigation of pyrvinium and other FDA-approved compounds that suppress Wnt/β-catenin signaling in human breast cancers is required to determine whether these therapeutics improve ER^+^ breast cancer outcomes. Furthermore, testing of potential combination therapies in non-cancerous *versus* preclinical breast cancer models would allow evaluation of on-target toxicity and efficacy of simultaneous *versus* sequential treatment regimens.

## PI3K signaling suppresses Wnt/β-catenin activation in ER^−^ breast cancer

There is significant evidence that primary human ER^−^ breast cancers exhibit enhanced Wnt/β-catenin signaling including increased β-catenin protein levels and Wnt gene expression [94,95,104,105]. In contrast with ER^+^ breast cancers, there is emerging evidence that class I PI3K signaling suppresses Wnt/β-catenin in ER^−^ breast cancers (summarized in Table 1). RNA-seq analysis of human ER^−^ breast cancer cells treated with buparlisib (PI3Kα/β/δ/γ inhibitor) revealed up-regulation of Wnt pathway gene expression including increased expression of Wnt ligands (*WNT3*, *WNT7B*, *WNT9A*, *WNT10B*), receptor complex proteins (*FZD1*, *FZD2*, *FZD4*, *FZD7*, *FZD9*, *LRP4*, *LRP6*, *PTK7*) and *CTNNB1* mRNA [106]. Furthermore, treatment of ER^−^ breast cancer cells with pictilisib, a strong PI3Kα/δ and modest PI3Kβ/γ inhibitor, increased mRNA expression of Wnt ligands including *WNT2B*, *WNT3*, *WNT5B* and *WNT10A*, as well as phosphorylation of LRP6 and β-catenin nuclear translocation [107]. Similar effects were also observed in canine mammary cancer cell lines that exhibit high basal Wnt/β-catenin activity, where treatment with dactolisib (dual pan-PI3Kα/γ/δ/β and mTOR inhibitor) enhanced TCF/LEF transcriptional activity, and *CTNNB1* and *AXIN2* mRNA expression [108]. Collectively, these findings suggest that class I PI3K and/or mTOR signaling suppresses Wnt/β-catenin signaling in ER^−^ breast cancer cell lines, although whether Wnt activation occurs in primary human ER^−^ breast tumors treated with PI3K inhibitors remains to be determined. The relative contribution of PI3Kα/β/γ/δ isoforms to Wnt/β-catenin down-regulation in ER^−^ breast cancer is also unknown and should be investigated in future studies using isoform-specific deletion or small molecule inhibition, particularly as PI3Kβ plays a critical role in *PTEN*-null ER^−^ breast cancer [50–53].

The mechanisms by which PI3K–Wnt crosstalk occurs in ER^−^ breast cancer are poorly understood. As mTOR suppresses FZD levels on the plasma membrane of intestinal stem cells [91], it is intriguing to consider whether PI3K/mTOR inhibition may increase FZD surface expression in ER^−^ breast cancer. In addition, buparlisib (PI3Kα/β/δ/γ inhibitor) treatment increased mRNA/protein expression of PORCN, an O-acetyltransferase required for Wnt ligand palmitoleoylation and secretion [106]. Therefore, class I PI3K down-regulation of Wnt ligand processing may also suppress Wnt/β-catenin signaling. It is also interesting to speculate that PI(4,5)P_2_ or PI(3,4,5)P_3_, the substrate and product of class I PI3K function respectively, may also contribute to Wnt suppression. For example, the PI(3,4,5)P_3_ effector BTK suppresses Wnt/β-catenin activation in human colorectal cancer and B cells by up-regulating the Wnt repressor, CDC73 [109]. In contrast, PI(4,5)P_2_ is required for Wnt/β-catenin activation by facilitating LRP phosphorylation and Dishevelled recruitment [110,111]. Further investigation is required to determine the molecular mechanisms of PI3K–Wnt crosstalk in this context.

The induction of Wnt/β-catenin signaling in ER^−^ breast cancers with PI3K/mTOR inhibition suggests that Wnt activation may reduce the sensitivity of these breast cancers to PI3K inhibitors. Pictilisib (strong PI3Kα/δ, modest PI3Kβ/γ inhibitor) treatment had little effect on the proliferation of ER^−^ breast cancer cells, whereas combined pictilisib and LGK-974 (PORCN inhibitor) treatment synergistically reduced ER^−^ breast cancer cell proliferation [107]. LGK-974 and buparlisib (PI3Kα/β/δ/γ inhibitor) treatment also synergistically reduced ER^−^ breast cancer cell viability and xenograft tumor growth [106]. Similar effects have been observed in other cancers such as pancreatic cancer, where combined ETC-159 (PORCN inhibitor) and pictilisib treatment synergistically reduced pancreatic cancer cell proliferation and xenograft tumor growth [112]. Recently, phase I clinical trials were conducted in individuals with metastatic ER^−^ breast cancer using the dual PI3Kα/γ and mTOR inhibitor, gedatolisib, in combination with cofetuzumab pelidotin, an antibody–drug conjugate with an auristatin payload targeting the Wnt co-receptor, protein tyrosine kinase 7 (PTK7) [113]. This PI3K/Wnt combination therapy had manageable toxicity, and some individuals exhibited a partial clinical response with disease stabilization [113]. However, further trials are required to assess whether PI3K/Wnt combination therapies can improve ER^−^ breast cancer outcomes.

## Conclusion and future directions

Class I PI3K signaling is one of the most frequently hyperactivated pathways in human breast cancer, and there have been significant advances in developing therapeutics that target this pathway in breast cancer. However, the clinical success of PI3K-targeted therapies remains limited, in part due to the complex interplay between PI3K and other signaling networks including estrogen or insulin. More recent findings show that PI3K signaling also undergoes crosstalk with the Wnt/β-catenin pathway with opposing effects observed in different breast cancer subsets. In *PIK3CA*-mutant ER^+^ breast cancer, PI3Kα signaling promotes INPP4B-dependent lysosomal degradation of GSK3β, which enhances Wnt/β-catenin activation. Conversely, in ER^−^ breast cancer, inhibition of class I PI3K and/or mTOR leads to Wnt/β-catenin activation. However, the molecular mechanisms and the contribution of individual PI3K isoforms are unknown. This may be influenced by the genetic or epigenetic profile of these distinct breast cancer subsets, such as the presence of *PIK3CA* mutations and increased INPP4B, or the expression of effectors that mediate PI3K suppression of Wnt signaling.

Although animal models have improved our understanding of cancer biology, there are limitations in extrapolating results from these model systems to humans. Human tumor organoids and patient-derived tumor xenografts are powerful, preclinical models for mechanistic and therapeutic testing with potential to circumvent the limitations of mouse models. Examination of PI3Kα, β, δ, or γ inactivation in normal and ER^+^ *versus* ER^−^ human breast cancer 3D organoids would help elucidate whether specific PI3K isoforms elicit opposing effects on Wnt signaling in preclinical models. Integrating Wnt/β-catenin reporter assays with PI3K signaling effector activity could provide further clarification of the mechanistic differences in PI3K and Wnt/β-catenin crosstalk in different breast cancer subtypes. However, comparing Wnt/β-catenin activation across different breast cancer subtypes can be challenging. Nuclear β-catenin localization is not evident across all subtypes [94,95] and β-catenin transcriptional reporter assays require co-expression of multiple probes that can be challenging in preclinical models. This may be improved by the development of quantitative live cell imaging biosensors to examine effector activation across subcellular compartments, such as those described for β-catenin or AKT [114,115]. Furthermore, studies of human model systems would benefit from comparison of PI3K–Wnt crosstalk in stem *versus* non-stem breast cancer cell states which exhibit distinct differences in PI3K and Wnt signaling mechanisms [78,116] (reviewed in [117,118]).

Wnt-targeted therapies may be a credible therapeutic strategy for treating ER^+^ or ER^−^ breast cancers. Although these therapeutics are yet to be evaluated in human breast cancer trials, data obtained from cell culture and mouse models suggests ER^+^ breast cancers with *PIK3CA*-mutations and/or increased INPP4B expression may benefit from Wnt therapies in combination with current standard-of-care treatments, whereas ER^−^ breast cancers may respond to combination therapy of Wnt and PI3K/mTOR inhibition to prevent Wnt reactivation. There has been substantial interest in developing Wnt therapeutics including small molecule inhibitors, peptides and antibodies that suppress Wnt/β-catenin signaling [119]. However, most Wnt-targeted inhibitors elicit significant adverse effects and/or do not effectively suppress Wnt/β-catenin signaling in human tumors, and there are currently no approved Wnt inhibitors for clinical use. Phase I clinical trials with the antibody–drug conjugate cofetuzumab pelidotin suggest this treatment exhibits partial efficacy with limited toxicity in ER^−^ breast cancers in combination with gedatolisib [113]. The repurposing of existing FDA-approved NSAIDs and anti-parasitic drugs that suppress Wnt/β-catenin signaling is emerging as a more promising clinical strategy [120], such as pyrvinium which was selectively cytotoxic to INPP4B-overexpressing ER+ breast cancer cells in combination with 4-OHT [102]. Further investigation of Wnt-targeted therapies in preclinical and clinical models, including careful assessment of the efficacy and on-target toxicity of these therapeutics in cancerous *versus* non-cancerous models will help to elucidate whether targeting the Wnt pathway is a viable therapeutic strategy for human breast cancers.

## Perspectives

- Class I PI3K signaling is one of the most frequently hyperactivated pathways in breast cancer, and its crosstalk with other signaling pathways contributes to the limited efficacy of PI3K-targeted therapeutics.
- Recent findings show that PI3K signaling enhances Wnt/β-catenin signaling in ER^+^ breast cancer by promoting GSK3β lysosomal degradation, but paradoxically suppresses Wnt/β-catenin activation in ER^−^ breast cancer by unknown mechanisms.
- Understanding the complex interplay between PI3K and Wnt signaling in breast cancer will elucidate novel mechanisms of crosstalk and determine whether Wnt-targeted therapeutics will play a future role in breast cancer treatment.

## Open Access

Open access for this article was enabled by the participation of Monash University in an all-inclusive *Read & Publish* agreement with Portland Press and the Biochemical Society under a transformative agreement with CAUL.

## Abbreviations

APC: adenomatous polyposis coli
ARF GEF: ADP ribosylation factor guanine nucleotide exchange factor
ARNO: ARF nucleotide-binding site opener
BTK: Bruton's tyrosine kinase
CK1α: casein kinase 1 alpha
EGFR: epidermal growth factor receptor
ER: estrogen receptor
ESCRT: endosomal sorting complex required for transport
FZD: frizzled
GRP-1: general receptor for phosphoinositides 1
GSK3β: glycogen synthase kinase 3 beta
HER2: human epidermal growth factor receptor 2
HRS: hepatocyte growth factor-regulated tyrosine kinase substrate
INPP4B: inositol polyphosphate 4-phosphatase type II
LEF: lymphoid enhancer-binding factor
LOH: loss of heterozygosity
LRP5/6: low density lipoprotein receptor-related protein 5/6
mTORC2: mammalian target of rapamycin complex 2
PDK1: phosphoinositide-dependent kinase 1
PH: pleckstrin homology
PI(3)P: phosphatidylinositol 3-phosphate
PI(3,4)P_2_: phosphatidylinositol 3,4-bisphosphate
PI(3,4,5)P_3_: phosphatidylinositol 3,4,5-trisphosphate
PI(3,5)P_2_: phosphatidylinositol 3,5-bisphosphate
PI(4,5)P_2_: phosphatidylinositol 4,5-bisphosphate
PI3K: phosphoinositide 3-kinase
PIPP: proline-rich inositol polyphosphate 5-phosphatase
PORCN: porcupine O-acyltransferase
PP2A: protein phosphatase 2A
P-Rex1: phosphatidylinositol-3,4,5-trisphosphate dependent Rac exchange factor 1
PTEN: phosphatase and tensin homolog
PTK7: protein tyrosine kinase 7
RTK: receptor tyrosine kinase
SERD: selective estrogen receptor degrader
SGK3: serum and glucocorticoid-regulated kinase 3
SH2: Src homology 2
SNX2: sorting nexin 2
TAPP1/2: tandem PH domain-containing proteins 1/2
TCF: T-cell factor
β-TrCP: beta-transducin repeat-containing protein
